# A Silent and Chronic Complication of Percutaneous Endoscopic Gastrostomy Tube: Small Bowel Enterocutaneous Fistula

**DOI:** 10.1155/2016/5328240

**Published:** 2016-11-06

**Authors:** Sai Wah Cheung

**Affiliations:** Division of Gastroenterology and Hepatology, Department of Medicine and Geriatrics, Tuen Mun Hospital, Tsing Chung Koon Road, Tuen Mun, New Territories, Hong Kong

## Abstract

Percutaneous endoscopic gastrostomy (PEG) has gradually gained the popularity since its invention and become the most preferred method for gastrostomy insertion in recent years. PEG is associated with lower morbidity and mortality and has the advantages of being minimally invasive and more convenient over the conventional open gastrostomy. However, significant rates of major complication still occur. Enterocutaneous fistula is one of the key complications that can be easily neglected due to its asymptomatic nature. We present a case of small bowel enterocutaneous fistula which was only found 8 years after the PEG insertion, being diagnosed after the longest duration of delay in diagnosis reported in literature.

## 1. Introduction

Percutaneous endoscopic gastrostomy (PEG) has dramatically renovated the practice in gastrostomy placement since it was first described in by Gauderer et al. at the University of Cleveland in 1980 [1]. Among the patients requiring long-term enteral feeding due to various reasons, it has become the most popular procedure in recent years due to the minimal invasiveness, economic cost, and ease to perform compared to traditional surgical gastrostomy. Despite the high safety profile of this technique, there are still significant perioperative complication and morbility rate of 3%–17.5% according to different series [2–6]. We hereby report a patient suffering from a rare complication of small bowel enterocutaneous fistula in a longstanding PEG whereas the patient remained completely asymptomatic for the longest duration that has ever been reported.

## 2. Case Presentation

A 35-year-old female patient suffering from severe mental retardation and epilepsy since birth was a long-term resident of the mentally incapacitated unit of the hospital. She was bedridden with a small body build of a weight of 32 kg. She had a PEG insertion done 8 years ago and then received regular PEG tube change every 3 months by the nurses and maintained well on the gastrostomy bolus milk feeding. Her body weight was static over the past years and there was no sign or symptoms of malnutrition or diarrhea.

**Figure 1:**
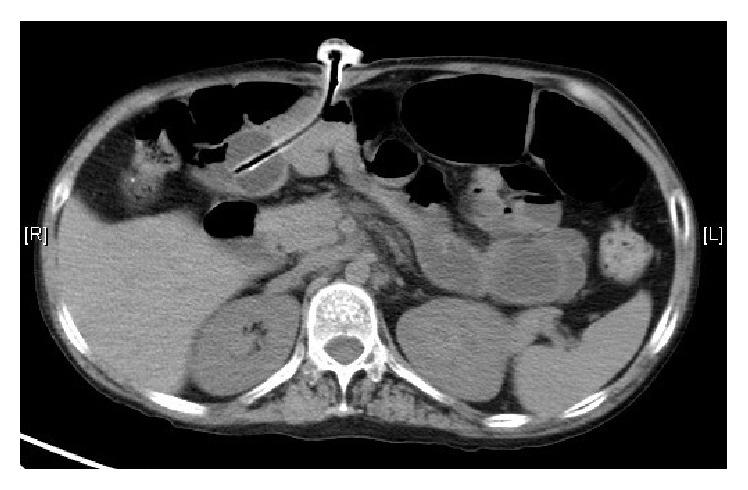
Computer topography of the abdomen showing the PEG tube extending over the right in the abdominal cavity and the opening in close proximity to the jejunum.

She presented to our gastroenterology unit for failure to deflate the PEG balloon during a regular PEG exchange due to blockage of the inflation/deflation catheter. Upon admission, the physical examination of the abdomen was normal with active bowel sound and negative for any distension or tenderness. The routine blood tests including complete blood picture, electrolytes, and liver and renal function tests did not review any abnormality. An oesophagogastroduodenoscopy (OGD) was arranged with an attempt to examine and deflate the balloon in the stomach endoscopically. An OGD examination failed to identify any gastrostomy tube in the upper gastrointestinal (GI) lumen with the scope passed down to the second part of the duodenum. An urgent computer tomography (CT) of the abdomen revealed the distal end of the PEG tube was likely situated at the proximal jejunum distal to the duodenojejunal junction with a normal fill-filled balloon intraluminally. There was no radiological feature of pneumoperitoneum (Figure 1). The PEG inflated balloon was finally identified endoscopically in the jejunum at 160 cm of the upper GI tract by a push enteroscopy and it was then punctured with an endoscopic needle knife. The PEG tube was successfully removed after the release of the internal bumper.

A nasogastric tube was inserted and used as a temporary measure for nutritional support and the fistula was allowed to heal spontaneously. A laparoscopic gastrostomy insertion was arranged for the patient. Intraoperatively, the previous PEG site at the jejunum was found to be adhered to the antrum of the stomach and the anterior abdominal wall. Another new gastrostomy tube was inserted by the operation and the patient tolerated gastrostomy feeding well afterwards.

## 3. Discussion

Since its innovation in the early 1980s, PEG has gained the popularity in gastrostomy insertion over laparoscopic gastrostomy as it avoids the need of general anesthesia and can be performed much less invasively. Overall it carries a successful rate as high as 98% and a lower risk of mortality compared to open gastrostomy of less than 1% [2, 3, 5, 7], and the procedure was also shown to be convenient and feasible even in an outpatient setting [8]. Despite its exceptional safety profile, perioperative complications including oesophageal tear, wound infection, external migration of the inner flange, colonic interposition or perforation, and peritonitis still happen from 2.3% to 17.5% according to different series [2, 3, 5–7].

Enterocutaneous fistula occurs when a segment of small or large bowel entrapped between the stomach and the abdominal wall. It is highly unusual in adults; a large case cohort identified only 6 cases of colocutaneous fistula out of 2384 adult patients receiving PEG insertion [9] while another single-centre experience retrospectively reported a complication rate of 0.76% [10]. However, it appears to be significantly more common in pediatric case series with a reported rate up to 3.5% [11, 12]. It is likely that the small size of the stomach causes limited apposition between the abdominal wall and the anterior wall of stomach and thus the chance of needle puncture through the colon becomes higher during the operation. Also, in paediatric population, the stomach rotates more easily upon inflation pulling the transverse colon in front of the stomach [13]. In our patient, although she received the gastrostomy at an adult age of 27 years, her body weight of 32 kg and lack of muscle mass made her mimicking a paediatric body build and rendered her to be at a higher risk than a usual adult. Other risk factors in previous reports include spinal kyphoscoliosis, prior abdominal surgery and adhesion, and overinflation of the stomach causing anterior rotation of the greater curvature of the stomach [10, 11, 14, 15].

A proper technique of transillumination, finger indentation of the ventral abdominal wall, and observation of any gas bubble aspirated from the injecting needle before entrance into the stomach during the operation can minimize the risk of interposition of the colon or small bowel [5, 10, 15]. In our patient, the internal bumper of the gastrostomy tube was initially confirmed to be in situ at the time of insertion 8 years ago. It is not certain when the feeding end of the tube slipped out from the stomach and lodged in the jejunum but probably this happened at the time of regular exchange. It is fortunate that the patient did not develop any peritonitis from the fistula tract and remained well on the tube feeding. Traditionally, open laparotomy was employed to positionally confirm or remove the misplaced tube [10, 11, 13, 16]. Nevertheless, with the current advances in the deep enteroscopy, it is likely that most misplaced PEG could be identified endoscopically and removed through enteroscope-assisted technique even if the internal bumper is fixed deep in the small bowel and the use of this technique avoids the need for a major surgery in a misfed and undernourished patient.

The majority of the enterocutaneous fistula resulting from PEG insertion reported from the literature is colocutaneous fistula and most of them would be symptomatic. The presentation includes diarrhoea upon tube feeding, faecal discharge from the tube, weight loss, abdominal pain, intestinal obstruction, and difficulty in tube exchange and also acute peritonitis after procedure [6, 9, 11, 12, 15]. The time from PEG to symptom presentation or diagnosis ranges from the period immediate after insertion to a longest 3 years of duration [9, 11, 16]. Enterocutaneous fistula, on the other hand, is much more infrequent. To date, only 3 cases of small bowel fistula created through endoscopic gastrostomy insertion were described in English literature [13, 15, 17]. In contrast to colocutaneous fistula, enterocutaneous fistula is often less symptomatic and usually only presents as mild bilious fluid leakage around the gastrostomy tube or even being completely asymptomatic. The appearance of symptoms occurred from 3 weeks to 14 months after the insertion in these cases. Our case is unique in that she was entirely asymptomatic for 8 years and even being asymptomatic at the time of diagnosis.

In conclusion, small bowel enterocutaneous fistula is a rare complication of PEG tube placement which can remain silent, asymptomatic for many years, and neglected easily. Careful case selection to avoid procedure candidate with multiple high risk factors and proper endoscopic techniques are useful in avoiding this complication. Moreover, the healthcare workers responsible for the PEG care have to take caution on any gastrointestinal, abdominal, or tube-related symptoms of the patients to ensure an early diagnosis and management.
